# Electronic medical oxygen dashboard system for daily oxygen monitoring and stockout prevention in Lesotho

**DOI:** 10.5588/pha.24.0046

**Published:** 2025-03-01

**Authors:** K.S. Joseph, T. Ntelane, T.D. Lekhela, M. Mungati, M. Shoba, S. Montsi, S.F. Leluma, L. Oyewusi, H. Schuh, B. Hansoti, J. Mirembe, N. Shilkofski, M. Strachan, N. Mahachi, E.D. McCollum

**Affiliations:** ^1^ Global Program for Pediatric Respiratory Sciences, Eudowood Division of Pediatric Respiratory Sciences, Pediatrics, and; ^2^ Department of Anesthesiology and Critical Care Medicine, Johns Hopkins University School of Medicine, Baltimore, MD, USA;; ^3^ Jhpiego Lesotho, Maseru, Lesotho;; ^4^ Department of Epidemiology, Johns Hopkins Bloomberg School of Public Health, Baltimore, MD, USA;; ^5^ Department of Emergency Medicine, Johns Hopkins University School of Medicine, Baltimore, MD, USA;; ^6^ United States Agency for International Development, Maseru, Lesotho;; ^7^ Jhpiego, Baltimore, MD, USA.

**Keywords:** developing countries, Lesotho, Africa, hypoxia, respiratory tract infections, oxygen

## Abstract

An electronic dashboard for oxygen monitoring and stockout prevention was implemented during the COVID-19 pandemic in 2022 by Jhpiego through the United States Agency for International Development Reaching Impact, Saturation, and Epidemic Control program and Government of Lesotho across 12 hospitals in Lesotho. Nurses documented patient blood oxygen levels, oxygen usage, and facility-level stocks on a daily checklist, which populated a dashboard that estimated oxygen demand usage and visualized facility-level oxygen stocks and impending stockouts. During 359 facility days evaluated, dashboard data reported 82/1,796 patients (4.6%) as hypoxemic, 186,802 L/day average oxygen usage, and successful prevention of all 14 potential stockouts.

## BACKGROUND

### Medical oxygen in low- and middle-income countries

Oxygen is an essential medicine for hypoxemia (low blood oxygen levels).^1^ Identifying and treating hypoxemia with oxygen is critical for reducing global mortality from many diseases.^2^ While data suggests that the hypoxemia burden is large and of greatest magnitude in low and middle-income countries (LMICs),^3^ oxygen access is limited in LMICs.^4,5^

### ‘Hub-and-spoke’ oxygen system

Hypoxemia and oxygen demand fluctuate depending on the interplay between circulating respiratory pathogens, air pollutant levels, and other factors like the underlying population’s risk profile. Thus, oxygen supply and demand at individual facilities are at constant risk of imbalance, particularly in settings with immature oxygen systems. When one healthcare facility has an adequate oxygen supply, another facility may be experiencing a respiratory viral surge, depleting oxygen resources.

A ‘hub-and-spoke’ oxygen system has oxygen production ‘hubs’ that fill oxygen cylinders and distribute them to a network of facilities (‘spokes’).^6^ Compared to more resource-intensive systems, a ‘hub-and-spoke’ system may have the greatest likelihood of sustainability in LMICs as it does not rely on facilities to produce their oxygen supply.^6^ Because facilities instead depend on more centralized oxygen production and delivery, a facility-based oxygen monitoring system is key for projecting oxygen demands while tracking stock and usage, allowing for stable planning of adequate oxygen stores. Crucially, a ‘hub-and-spoke’ system must be responsive to hypoxemia surges to be effective. Reliably estimating hypoxemia requires routine use of pulse oximeters, devices that non-invasively measure blood oxygen levels (peripheral arterial oxyhemoglobin saturation [SpO_2_]). These elements underpin an oxygen system’s primary goal, optimizing oxygen access while avoiding stockouts and related catastrophic patient outcomes. To date, few health systems in LMICs have reported such oxygen systems.^7^

### COVID-19 pandemic and Lesotho: an emergency ‘hub-and-spoke’ oxygen system

The COVID-19 pandemic brought unparalleled attention to medical oxygen, its scarcity in LMICs, and the extreme vulnerability of hypoxemic patients.^5^ It also provided a unique opportunity to generate long overdue momentum towards strengthening LMIC oxygen systems. In Lesotho, a national COVID-19 readiness assessment conducted in 2020 revealed stark limitations in hypoxemia identification and oxygen availability nationally.^8^ As part of Lesotho’s COVID-19 emergency response, substantial investments and efforts were mobilized to address these deficits by the United States Agency for International Development Reaching Impact, Saturation, and Epidemic Control program through Jhpiego and in partnership with the Government of Lesotho, aiming to strengthen hypoxemia monitoring, oxygen usage, and bolster facility-level oxygen supplies as a stronger and more structured ‘hub-and-spoke’ oxygen system was conceived. As of early 2022, the two national COVID-19 treatment centers in Lesotho had pressure swing adsorption (PSA) oxygen production plants installed to deliver piped oxygen. In contrast, other hospitals in Lesotho without oxygen production capacity relied on oxygen concentrators and free-standing portable oxygen cylinders produced and distributed from these PSA plants or international vendors. In response to the pandemic, frequent oxygen shortages, poor patient outcomes, and the lack of an oxygen monitoring system, Jhpiego coordinated with the Government of Lesotho to create an emergency oxygen distribution ‘hub’ and an electronic oxygen dashboard monitoring system to support oxygen distribution to hospitals. The ‘hub’ included one healthcare provider and vehicle tasked with coordinating and distributing filled oxygen cylinders to facilities. The dashboard system was developed and piloted in late 2021 and implemented in February 2022 in 12 hospitals, including COVID-19 treatment centers and 10 district hospitals. The dashboard estimated oxygen demand usage and visualized facility-level oxygen stocks and imminent stockouts.

### Oxygen monitoring: dashboard system inputs

One nurse at each hospital was oriented to the system and designated as the facility oxygen dashboard provider. During regular work hours, each nurse used pulse oximeters to measure the SpO_2_ from hospitalized patients while breathing in room air and classified patients as hypoxemic (SpO_2_ < 94%) per Lesotho National COVID-19 guidelines.^9^ After measuring the SpO_2_, nurses counted filled and unfilled oxygen cylinders and functioning oxygen concentrators daily using a pragmatic checklist (without identifiable patient information) and categorized cylinders in use with patients into flow ranges (1–5, 6–9, 10–13 and 14–17 L/minute [LPM]). As adults using concentrators typically required 5–10 LPM, a 7.5 LPM flow was used for concentrators. Subsequently, nurses scanned the checklist onto a phone, sharing deidentified data with a centralized data clerk. Data were entered into a DHIS2 system, and Microsoft PowerBi software modeled and visualized the data on the electronic dashboard. Oxygen stock volumes were calculated by summing unused oxygen (filled cylinder volumes and functioning concentrator capacities). Daily oxygen usage volumes were estimated from median LPM values from each flow category.

### Oxygen monitoring: dashboard system outputs

The dashboard presented daily oxygen stocks (filled and unfilled cylinders, in-use and total concentrators) and estimated the days remaining of oxygen stock according to the current usage rate by the facility. If the automated algorithm considering oxygen usage and stocks predicted oxygen shortages within three days, the dashboard would highlight the facility as red, prompting the distribution ‘hub’ to transfer cylinders to the facility. Daily dashboard visualization was electronically shared with the distribution ‘hub,’ participating hospitals, the Ministry of Health, and other stakeholders. During a 6-week evaluation period from February 7 to March 18, 2022, dashboard data estimated the national hypoxemia prevalence as 4.6% (82/1,796 patients) (Table). Dashboard data also showed that the total oxygen volume utilized was 7,472,080 L nationally (an average of 186,802 L/day), with 40% (2,990,080 L) supplied by cylinders and 60% (4,482,000 L) by concentrators. Notably, the dashboard identified 14 potential oxygen stockouts (3.9% of 359 days of facility-level surveillance) and reported 58% (7/12) of monitored hospitals as at risk of >1 stockouts. In each case, the distribution ‘hub’ was engaged, and the vehicle was able to successfully relocate filled cylinders to at-risk facilities, avoiding any outages (Figure).

**TABLE.** Lesotho medical oxygen dashboard system: oxygen demand and usage estimates during 2022 evaluation period^*^

Hospitals (*n* =12)	Oxygen demand			Oxygen usage		
	SpO_2_ measures	Hypoxemia^†^	Proportion of hypoxemia %	Total oxygen volume used L (95% CI)	Oxygen volume used in L/day mean ± SD	Peak daily oxygen use L
Total	1,796	82	4.6	7,472,080 (5,987,993–8,956,167)	186,802 ± 116,011	370,000
Berea^‡^	30	22	73.3	2,718,720 (2,135,432–3,302,008)	67,968 ± 45,595	158,400
Botha Bothe	243	6	2.4	345,600 (248,266–442,933)	11,917 ± 8,823	32,400
Machabeng	121	3	2.4	111,600 (13,327–209,872)	3,985 ± 9,051	32,400
Mafeteng^‡^	3	1	33.3	1,216,800 (945,044–1,488,555)	48,672 ± 26,344	97,200
Maluti	144	2	1.3	277,200 (139,882–414,517)	9,900 ± 12,647	50,400
Mokhotlong	168	5	2.9	442,800 (312,098–573,502)	15,268 ± 11,848	43,200
Motebang	150	5	3.3	1,371,600 (1,051,174–1,692,026)	52,753 ± 30,512	140,400
Ntsekhe	172	8	4.6	380,800 (224,842–536,757)	15,866 ± 15,389	60,400
Paray	142	12	8.4	241,200 (110,471–371,928)	17,228 (16,172)	43,200
Quthing	128	4	3.1	118,800 (38,447–199,152)	4,242 (7,400)	32,400
Saint Joseph	253	10	3.9	147,600 (16,125–279,074)	5,676 (12,519)	50,400
Scott	242	4	0.8	99,360 (15,275–183,444)	3,548 (7,744)	38,160

* Evaluation period from February 7 to March 18, 2022, includes data from 359 facility days.

† Hypoxemia defined as a SpO_2_ < 94% measured in room air.

‡ National COVID-19 Treatment Center Hospital utilized for confirmed or probable severe COVID-19 cases.

SpO_2_ = peripheral arterial oxyhemoglobin saturation; CI = confidence interval; SD = standard deviation.

**FIGURE.**
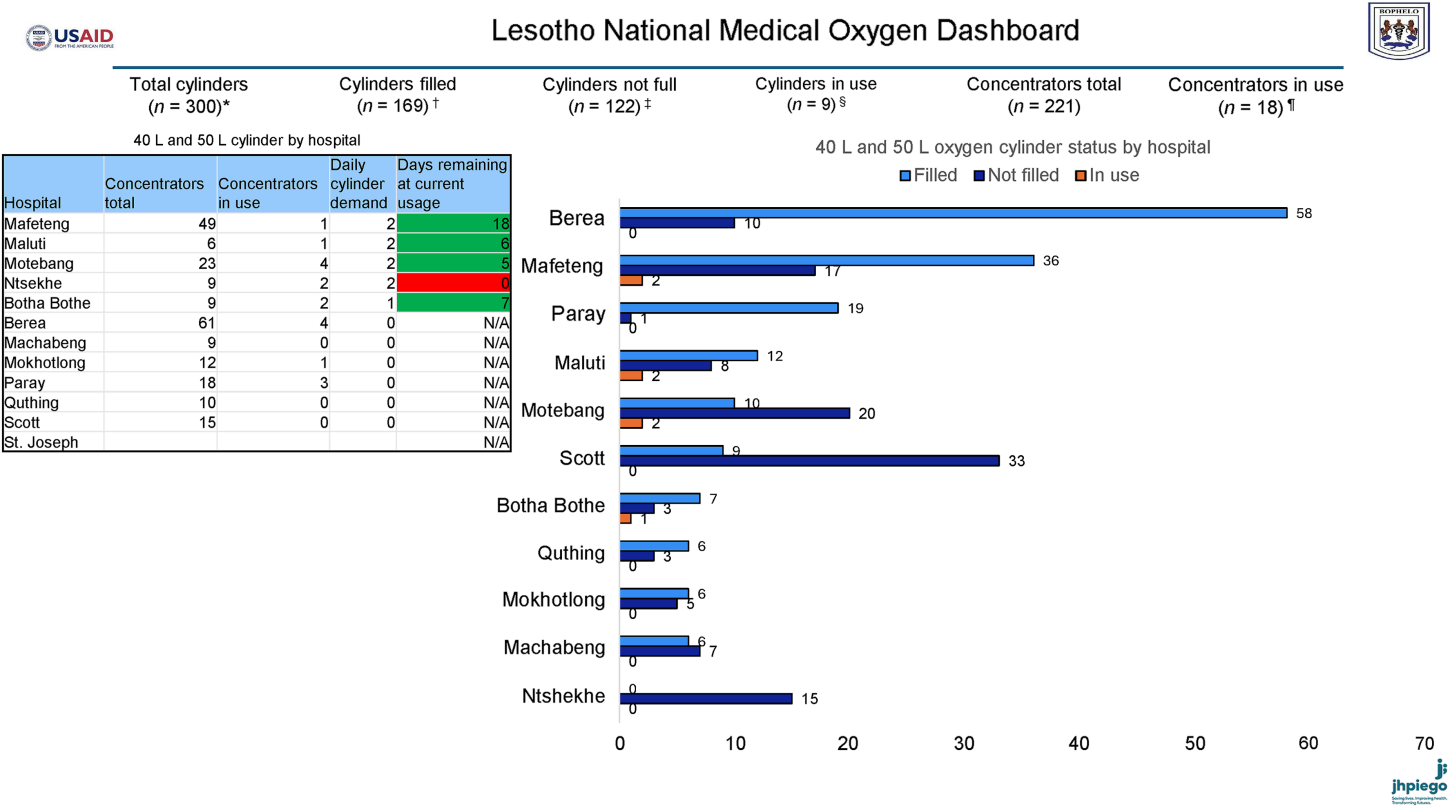
Lesotho national medical oxygen dashboard. Disclaimer: Oxygen cylinder information is dynamic and can greatly fluctuate based on patient use. The estimation assumptions used are conservative and, therefore, may underestimate the current oxygen supply. This report reflects the oxygen cylinder status as of 9 am each day from the reporting hospital. 50 L (7,700 L oxygen gas) and 50 L (5,600 L oxygen gas) cylinder sizes are counted. Smaller cylinders are not counted. Data were collected and shared by 9 am daily from Monday to Friday by the Ministry of Health and case management focal nurses. Indicators: *total cylinders: sum of filled, not full, and cylinders in use (40 L and 50 L sizes); ^†^cylinders filled: unopened cylinders (40 L and 50 L sizes); ^‡^cylinders not full: opened cylinders (40 L and 50 L sizes); ^§^cylinders in use: cylinders open and providing oxygen to patients (40 L and 50 L sizes); ^¶^concentrators in use: concentrators providing oxygen to patients; daily cylinder demand: sum of the flow rates of cylinders in use projected over 24–72 hours; days remaining at current usage: calculated estimate derived from the total cylinders filled and daily use.

In sum, this is a pragmatic, electronic national oxygen dashboard system premised on transparent, timely responsiveness to the daily fluctuating oxygen needs of patients and facilities. Our experience shows that a ‘hub-and-spoke’ oxygen system – when supported by an actionable monitoring and distribution network – can effectively support the care of hypoxemic patients in an African LMIC.
